# The association of blood ctDNA levels to mutations of marker genes in colorectal cancer

**DOI:** 10.1002/cnr2.1782

**Published:** 2023-02-06

**Authors:** Fei Bai, Qian Du, Qingliang Zou, Lin Xu, Wei Dong, Xinlin Lv, Xiaorong Han, Huijun Zhou, Chi Zhang, Tao Lu

**Affiliations:** ^1^ Hunan Cancer Hospital and The Affiliated cancer Hospital of Xiangya School of Medicine Central South University Changsha China; ^2^ School of Biological Sciences University of Nebraska Lincoln Nebraska USA; ^3^ Chengdu Medical College Chengdu Sichuan China; ^4^ Sichuan Cancer Hospital and Institute, Sichuan Cancer Center, School of Medicine University of Electronic Science and Technology of China Chengdu Sichuan China; ^5^ Department of oncology Chengdu Ping‐An Hospital Chengdu Sichuan China; ^6^ Chengdu Women and Children's Center Hospital Chengdu Sichuan China

**Keywords:** association study, cell‐free circulating tumor DNAs (ctDNA), colorectal cancer (CRC), genomic variations, random forest

## Abstract

**Background:**

Colorectal cancer (CRC) is a deadly and commonly diagnosed cancer. Cell‐free circulating tumor DNAs (ctDNA) have been used in the diagnosis and treatment of CRC, but there are open questions about the relationship between ctDNAs and CRC. Although mutations of genes detected by ctDNA in CRC have been studied, the quantitative relationship between ctDNA mutations and ctDNA concentration has not been addressed.

**Aims:**

We hypothesized that there was an association between mutations of genes identified in ctDNAs and ctDNA concentration. His study examined this association in a population of CRC patients.

**Methods:**

In 85 CRC patients, we sampled 282 mutations in 36 genes and conducted an association study based on a Random forest model between mutations and ctDNA concentrations in all patients.

**Results:**

This association study showed that mutations on five genes, ALK, PMS2, KDR, MAP2K1, and MSH2, were associated with the ctDNA concentrations in CRC patients’ blood samples. Because ctDNA mutations correlate with ctDNA level, we can infer the tumor burden or tumor size from ctDNA mutations, as well as the survival time for prognosis.

**Conclusion:**

Our findings shed light on the associations between mutations of genes identified in ctDNAs and ctDNA concentration in the blood of CRC patients. This discovery provides information regarding the tumor burden or tumor size based on ctDNA mutations.

## 1 INTRODUCTION

Colorectal cancer (CRC) is the fourth most commonly diagnosed cancer but the third most deadly cancer in the world—more than two million new cases and one million deaths per year worldwide.^1^ In routine diagnosis, determining the genomic characteristics of CRCs is based on the DNA extracted from tumor tissues obtained by biopsy or surgical removal, which are all invasive methods. Usually, DNAs from such a tissue sample are not representative of the heterogeneity of the entire tumor because a limited number of cells from a local area were used. As a complement to tumor tissue genotyping, liquid biopsy allows minimally invasive detection of potential tumor‐specific mutations and molecular profiling of their dynamics. Measuring ctDNA also allows for quantitative and qualitative real‐time evaluation of body fluids. In addition, a liquid biopsy can be repeated at intervals to monitor response to treatment, the development of drug resistance, and the detection of relapse.^2^, ^3^ Overall, the identification of ctDNA has led to lots of research for its applications to detect early disease, relapse, response to therapy, and emerging drug resistance mechanisms.^4^

Liquid biopsy can be used for the noninvasive detection of cell‐free circulating tumor DNA (ctDNA) or circulating tumor cells. The study of ctDNA can be applied to CRC, such as the detection of minimal residual disease (MRD) and tracking clonal dynamics in response to targeted therapies.^5^ Existing studies suggest that the detection of ctDNA in patients with CRC depends on the extent of tumor volume.^6^ Many studies suggested that ctDNA level is associated with tumor burden.^7^, ^8^ Studies also found that the changes in tumor volume measured by CT imaging had a kind of association with the changes in ctDNA levels.^9^ Besides serving as a reliable marker of tumor burden, where changes in the volume of disease with treatment or disease progression, ctDNA also carries genetic information.^10^, ^11^ Previous studies showed that ctDNA analysis discovered genomic changes in genes *RAS*, *BRAF*, *ERBB2*, *MET*, and other tumor‐related genes associated with resistance to anti‐epidermal growth factor receptor (EGFR) therapy could have higher diagnostic accuracy.^12^ Besides, longitudinal monitoring of ctDNAs during anti‐epidermal growth factor receptor therapy revealed that genomic changes occurred as an acquired drug resistance mechanism of specific genes, mainly genes associated with mitogen‐activated protein kinase (MAPK) signaling pathways. ctDNA analysis can also identify predictive biomarkers of immunocheckpoint inhibitors,^7^ such as mismatch repair gene mutations, microsatellite instability high phenotypes, and tumor mutation burden.^13^, ^14^ A number of prospective clinical trials are underway to assess the impact of targeted drugs on genomic ctDNA changes, or to monitor ctDNA to explore drug resistance biomarkers.^15^

Liquid biopsy, including ctDNA and circulating tumor cells, has been used in the diagnosis and treatment of CRC. For example, Yang et al. collected ctDNA mutations and concentrations in 47 early and late CRC patients and analyzed them using target sequencing and a panel covering 50 cancer‐related genes. Thirty‐seven ctDNA mutations were found in 93.6% of all patients. The results showed that TP53, PIK3CA, APC, and EGFR were the most commonly mutated genes. ctDNA concentration in advanced patients was significantly higher than that in early patients, and increased ctDNA concentration was associated with increased tumor size.^16^ Previous work showed that the overall concordance rate between ctDNA and matched tissues was 77.2% (78/101).^8^ The data confirm that the amplitude‐based NGS system can sensitively detect mutant alleles in cell‐free DNA (cfDNA). These results suggest that ctDNA may be a novel diagnostic biomarker for monitoring mutation status and changes in tumor burden in patients with mCRC.^8^ Liquid biopsy by ctDNA sequencing has great potential for early detection and postoperative monitoring of CRC. DNA from colon cancer tissue is released into the blood more easily than DNA from rectal cancer tissue.^17^ As a sensitive biomarker, ctDNA shows great potential in monitoring the response to multiple treatment modalities and targeted therapies for CRC.^7^

The ctDNA level has a certain correlation with tumor burden with a range of correlation coefficient around 0.5–0.7.^7^, ^8^, ^18^ There must be many other factors that can affect the ctDNA level, such as mutations in cancer cell genomes. Although ctDNA mutations in CRC have been studied, the quantitative relationship between ctDNA mutations and ctDNA concentration has not been addressed. We have a hypothesis that there is an association between ctDNA mutations and ctDNA concentration. We sampled 282 mutations for 36 genes across 85 patients and conducted a statistical analysis of mutations and ctDNA concentrations of patients. The association study showed that mutations on five genes, *ALK*, *PMS2*, *KDR*, *MAP2K1*, and *MSH2*, are associated with the concentration of ctDNAs. The association studies between ctDNA levels in the blood to therapeutic response, tumor burden, or tumor size were conducted.^7^, ^8^, ^9^, ^18^ Therefore, because mutations in ctDNA have an association with ctDNA level, then, we can infer the tumor burden or tumor size from gene mutations in ctDNA, and even the survival time for prognosis. The large tumor burden and high ctDNA level indicate shorter overall survival.^19^, ^20^, ^21^ We found the association between gene mutations and ctDNA levels provides new insight into ctDNA study. It can give us some hints about the reason that caused the change in ctDNA levels.

## 2 RESULTS

This study recruited CRC patients from January 1 to August 31, 2019, at Hunan Cancer Hospital, Changsha, China, under an approved institutional review board (IRB) protocol number, KYJJ‐2020‐132. A total of 85 CRC patients with stages I–IV, N0–N2, no distant metastasis (M0), and detectable ctDNAs, were selected under the following criteria—patients had the sporadic form of CRC, did not have other basic diseases, have no infection, did not have other factors affecting plasma free nucleic acid concentration, such as no neo‐adjuvant treatment, chemotherapy, and/or radiation therapy. The diagnosis of CRC was based on the examination of hematoxylin and eosin (H&E)‐stained histopathological images by pathologists. The clinical information of patients, including tumor sizes and stages, is summarized in Table 1 and their details are listed in the Dataset S1.

**TABLE 1:** The summary of 85 patients

Characteristic, *N* = 85	
Age median (years) (range)	56.5 (29–80)
Gender, *N* (%)	
Male	60 (70.59)
Female	24 (28.24)
Missing data	1 (1.17)
Tumor size (cm)	
<1 × 1 × 1	27 (31.76)
>1 × 1 × 1	40 (47.06)
Missing data	18 (21.18)
NCCN stage, *N* (%)	
T	
Tx	1 (1.18)
Tis	22 (25.89)
T1	‐
T2	26 (30.59)
T3	18 (21.18)
T4	7 (8.24)
Missing data	11 (12.92)
N	
Nx	10 (11.76)
N0	17 (20.00)
N1	28 (32.94)
N2	12 (14.12)
Missing data	18 (21.18)
M	
M0	26 (30.59)
M1	36 (42.35)
Missing data	23 (27.06)
Sample type, *N* (%)	
Blood	85 (100.00)
Fresh tissue	26 (30.59)
Paraffin‐embeded tissue	59 (69.41)

**FIGURE 1:**
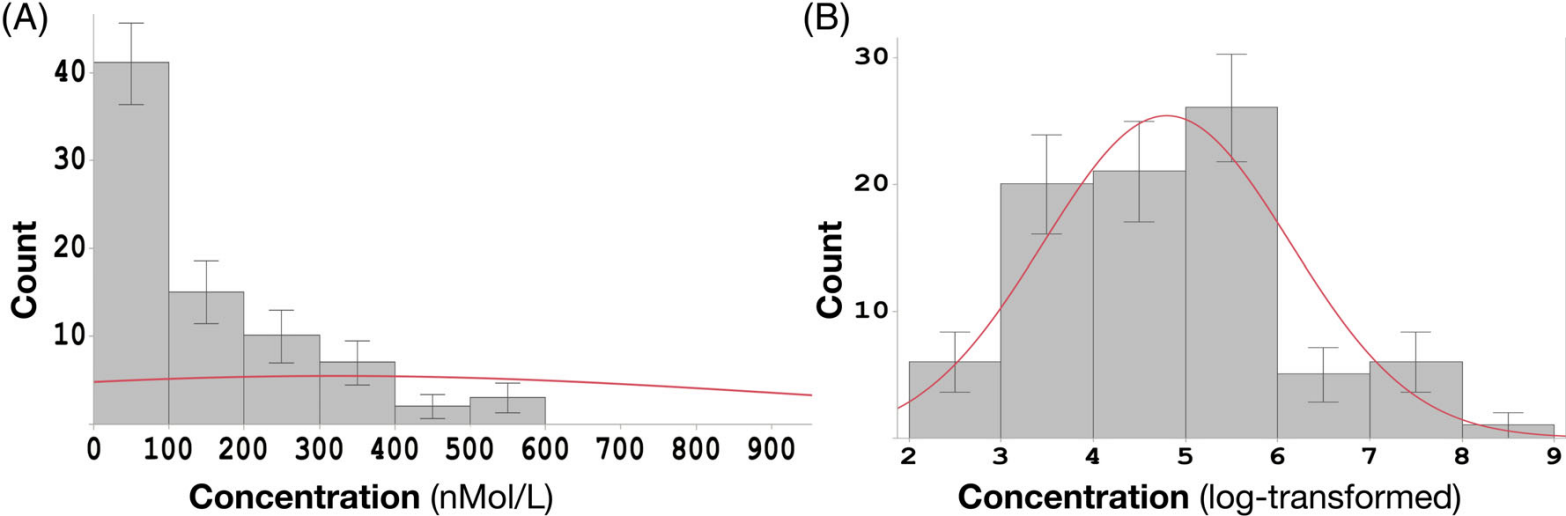
Distribution of ctDNA concentrations in all patients. (A) is the histogram of ctDNA concentration in the blood. (B) is the histogram after the logarithmic transformation. The red curve is the fitted normal distribution curve

Tumor tissues were collected at surgery, and blood samples (*n* = 85) were collected before surgery. Then, ctDNAs of 36 CRC marker genes were amplified and sequenced. Single nucleotide variants (SNVs) in these ctDNAs were called, and SNVs were validated by the corresponding DNAs obtained from tumor tissues. The ctDNA sequencing dataset has 282 mutations for 36 genes across 85 samples (Dataset S1). The objective is to identify mutations that could be used as a prognostic marker for ctDNA. The distribution of patient blood ctDNA levels is shown in Figure 1A. Most ctDNA concentrations are smaller than 100 ng/μl and the distribution is skewed. Therefore, we applied the log‐transform to all ctDNA concentrations (Figure 1B) to approximately conform to normality. Distribution fitting analysis showed the log‐transformed ctDNA values near a normal distribution.

**FIGURE 2:**
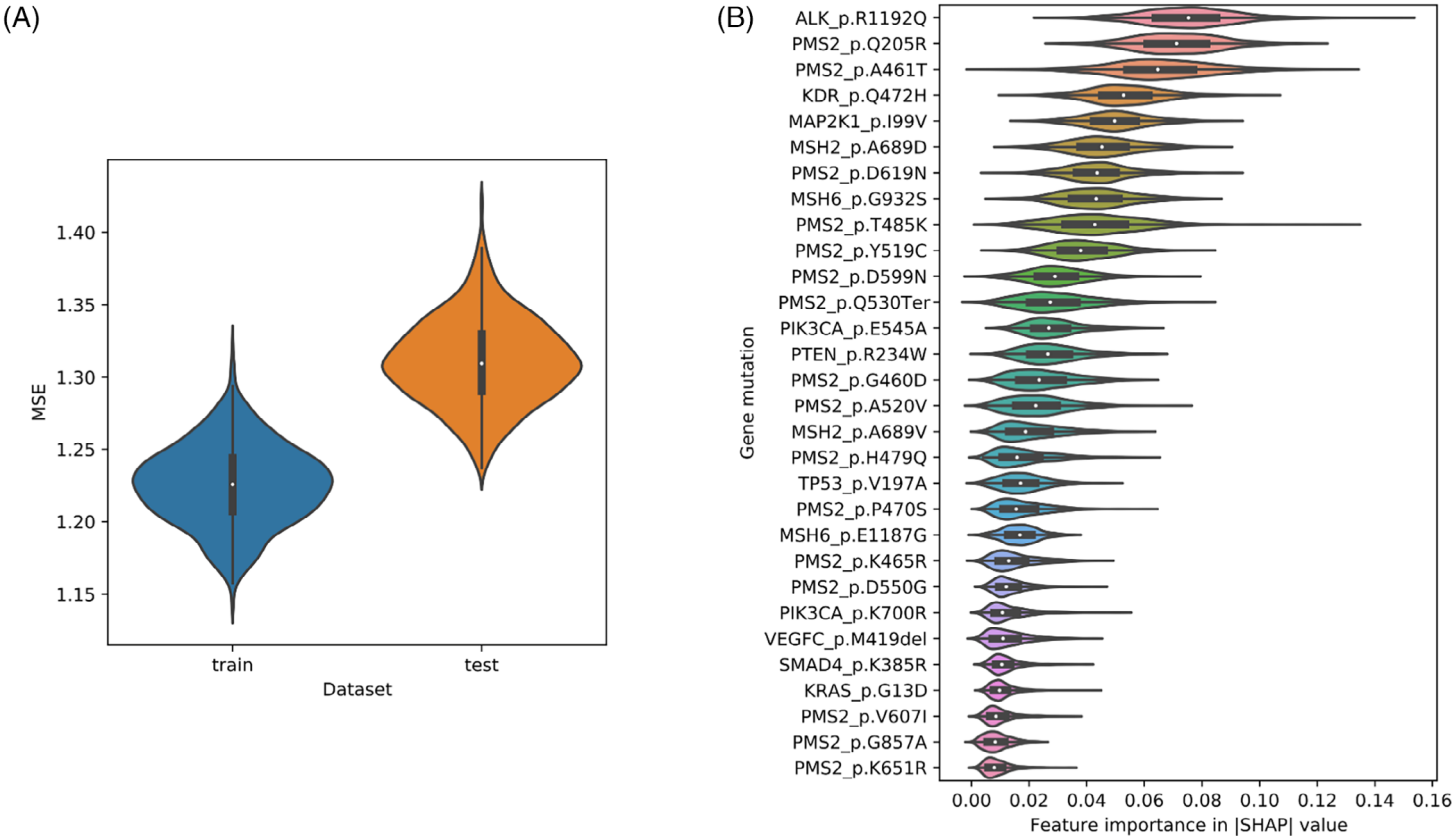
The prediction performance of the model evaluated by cross validation. (A) Violin plots for the distributions of MSE were calculated from the training procedure (blue) and the prediction procedure (orange) in all 1000 rounds. (B) Distribution of SHAP values of all mutations

**FIGURE 3:**
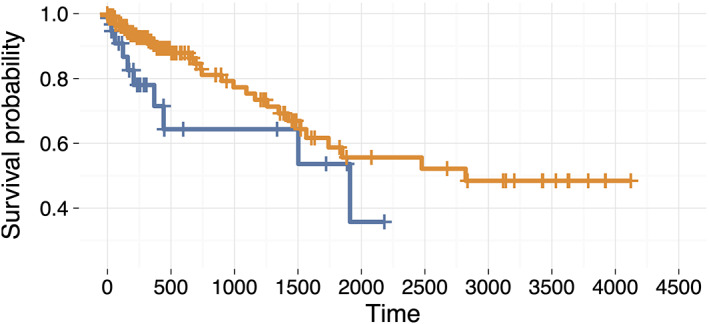
Survival curves of two categories of patients, with or without the gene mutations. The blue survival curve refers to the 76 TCGA colorectal cancer patients with mutations in at least one of the five genes (*ALK*, *PMS2*, *KDR*, *MAP2K1*, and *MSH2*), and the orange curve refers to the 411 patients without any mutations in the five genes

We used the random forest regression model to study the association between SNPs from tumor genes and ctDNA concentration. SNVs with minor allele frequency (MAF) < 0.01 were not considered to target SNPs only and final SNPs with correlation coefficient > 0.8 were grouped together. For the model building and validation steps, we split the whole dataset into a 60% training dataset and a 40% test dataset and repeated this step 1000 times for validation. For the Random Forest regression, we allowed the maximum features to be 5, that is, five mutations, and the number of independent trees to be 100. The average values and distribution of mean square errors (MSE) and SHAP values were calculated. Figure 2 shows the result for MSE. The average values of MSEs for the training and test datasets are 1.23 and 1.31, respectively. It is not surprising that the MSE for the test dataset is larger than that for the training dataset, but, compared to the distribution of ctDNA (Figure 1B), the value of the MSE is significantly smaller than all ctDNA values. This result indicates that there is a kind of association between tumor gene mutation and ctDNA concentration measures in the same patient. To understand the contribution of each gene mutation to the model, SHAP values,^22^ an alternative to permutation feature importance, were calculated to evaluate the importance of each gene mutation. Figure 3 shows the distribution of SHAP values for each gene mutation for 1000 round cross‐validations. ALK_p.R1192Q was ranked as the top feature for this prediction model based on the SHAP values. ALK_p.R1192Q has a negative correlation with the amount of ctDNA; this mutation appears in patients with relatively low ctDNA concentrations; 10 out of 11 patients with these mutations have ctDNA concentrations less than the average. Besides ALK_p.R1192Q, PMS2_p.Q205R, PMS2_p.A461T, KDR_p.Q472H, MAP2K1_p.I99V, and MSH2_p.A689D have large SHAP values in all gene mutations. All the rest five mutations have a positive correlation with the concentration of ctDNAs.

From the prediction model, mutations on five genes, *ALK*, *PMS2*, *KDR*, *MAP2K1*, and *MSH2*, (ALK_p.R1192Q, PMS2_p.Q205R, PMS2_p.A461T, KDR_p.Q472H, MAP2K1_p.I99V, and MSH2_p.A689D) are associated with the ctDNA level. The ctDNA level was not simply associated with tumor burden or dying cells, actually a complex reflection of tumor biology, but ctDNAs are considered as the result of apoptosis and necrotic cell death.^23^, ^24^ These discovered genes, mainly related to cell proliferation and death, could have a biological association with the biogenesis of ctDNAs or can work as biomarkers to the blood ctDNA level.

The large tumor burden or high ctDNA level has a certain association with shorter overall survival.^19^, ^20^, ^21^ We, therefore, tested if these five genes associated with the high ctDNA levels were associated with shorter overall survival. The CRC clinical data in TCGA were collected for survival analysis. Mutations in at least one of the five genes (*ALK*, *PMS2*, *KDR*, *MAP2K1*, and *MSH2*) were found in 76 out of the 487 patients in TCGA dataset. The survival analysis for 76 versus 411 patients was performed. There was a difference between these two groups of survival data, and the survival time of 76 patients with mutations was very shorter than the other 411 patients (Figure 3).

**FIGURE 4:**
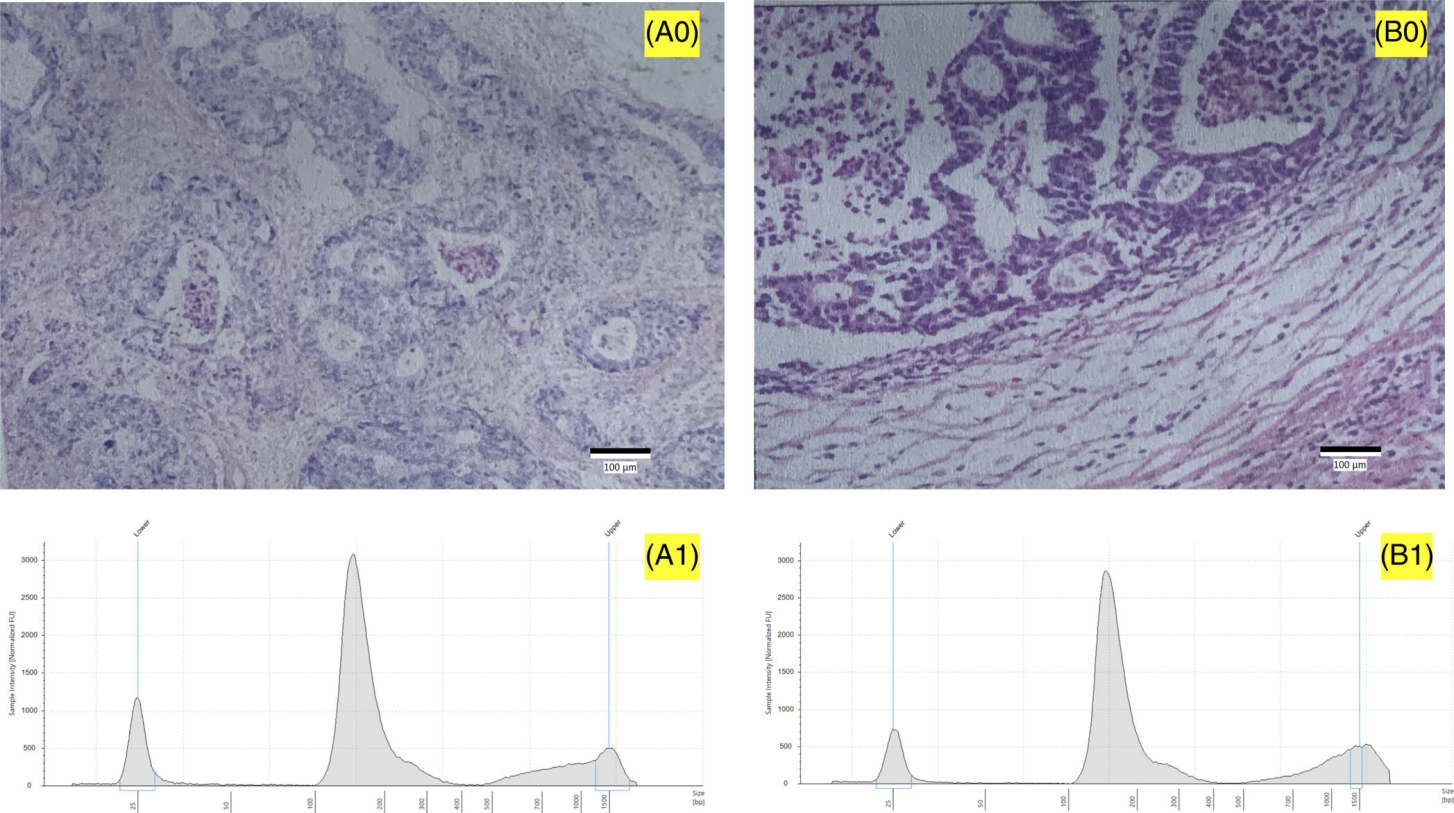
Patients' H&E‐stained histopathological images of tumor tissues and cfDNA concentrations. A0 and B0 were H&E‐stained histopathological images (magnification ×40) for patients P20190804_008 and P20190609_062. A1 and B1 are Agilent 4200 TapeStation images for cfDNAs from patients P20190804_008 and P20190609_062, respectively. In A1 and B1, the left and right outermost peaks are internal standards, while the middle peak of 200 bp is the cfDNA concentration

The patient P20190804_008 had mutations in the *PMS2* and *SMAD4* genes and high ctDNA concentration. This patient had ulcerated moderately differentiated adenocarcinoma with staging T3N2M1, and the tumor size is 3.5 × 3.0 × 2.2 cm with invasion to the serous membrane and parastomal lymphatic metastasis. The H&E‐stained histopathological image is shown in Figure 4. The patient's ctDNA concentration was up to 2300 nMol/L (Figure 4). At the same time, SMAD4_p.L364Ter mutation and 4 mutations in PMS2_p.A461T, PMS2_p.D550G, PMS2_p.P470S, PMS2_p.T485K were detected. The early termination of the SMAD4 gene product has an important effect on the abnormal elevation of ctDNA.

Another patient, P20190609_062, had mutations in the *KDR* gene and extremely high ctDNA levels. This patient had low‐differentiated adenocarcinoma with stage T3N0M0. His tumor size is 4.0 × 3.5 × 1.0 cm with invasion to the serous membrane. The H&E‐stained histopathological image is shown in Figure 4. Patient P20190609_062's ctDNA concentration was up to 1722 nMol/L (Figure 4B1). This patient had mutations that occurred in the *KDR* gene (KDR_p.Q472H) and in *PMS2* gene (PMS2_p.A461t, PMS2_p.P470s, PMS2_p.T485K). These mutations have large SHAP values in the statistical model.

## 3 DISCUSSION

Information on ctDNA has many potential applications, including early detection, monitoring for early recurrence, molecular profiling, and therapeutic response prediction.^4^ ctDNA may be used to inform clinical decision‐making using both tumor‐informed and tumor‐agnostic platforms.^4^ ctDNA has been studied in CRC, for example, the analysis of circulating tumor DNA to monitor disease burden following colorectal cancer surgery^25^ and the analysis of ctDNA in patients with stages I to III colorectal cancer.^26^ The ctDNA concentration itself can provide much useful information. For example, there is a hypothesis that ctDNA concentration during the therapy is associated with long‐term survival.^27^ There are ongoing studies to develop dynamic changes in ctDNA concentrations as a potential surrogate end‐point of clinical efficacy in patients undergoing adjuvant immunotherapy.^28^ Because the ctDNA concentration is useful in cancer studies, the association of the ctDNA concentration and tumor genomes is required to be investigated.

The level of ctDNA in the serum of individuals with cancer was higher than that of the healthy group.^29^ ctDNA carries the same specific mutations as the corresponding cancer cells, including single nucleotide mutations, structural mutations, and DNA methylation.^30^ A study showed that the ctDNA concentration in early‐stage patients was significantly lower than that in late‐stage patients and that the ctDNA concentration was positively correlated with tumor size.^16^ It has been discovered that tumor genomic mutations, such as SNV^31^ and copy number variants,^32^ have a correlation with tumor size or tumor progression. Therefore, it is logically reasonable that some mutations on ctDNAs have a certain level of association with the ctDNA concentration. The SNVs of ctDNAs associated with the high level ctDNA concentration indicates the larger tumor size and late stage. Interestingly, it has been discovered that, in CRC patients, changes in average variant allele frequency (VAF) in ctDNA showed a significant correlation with tumor size change.^33^ Therefore, gene mutations in ctDNA have the potential to serve as biomarkers for the tumor burden, tumor size, and even the survival time for prognosis. These facts agree with our findings.

Based on the association study between genomic variants and ctDNA concentration in 85 patients, it was revealed that mutations of genes *PMS2* and *ALK* were associated with blood ctDNA concentration levels. Other genes related to angiogenesis and regulation, such as *MAP2K1* and *KDR*, played a more important role in the high level of ctDNA concentration in blood. An elevation of ctDNA concentration in the blood is associated with proliferation, migration, tubule morphogenesis, and germination of neovascular endothelial cells, which are often related to tumor progression and migration. Therefore, through the quantitative analysis of ctDNA concentration, characteristics of tumors, such as marker genes and their mutations, generation, progression, and metastasis, can be found to a certain extent. Because these five genres, *ALK*, *PMS2*, *KDR*, *MAP2K1*, and *MSH2*, have important functions, CRC with somatic mutations on any of these genes showed worse survival rates than cases without somatic mutations (Figure 3). Due to the association between ctDNA level and somatic mutations of these genes, non‐invasive examination of semantic mutations of these genes via ctRNAs in patient blood has clinical application for prognosis.

*ALK* gene makes a protein called anaplastic lymphoma kinase (ALK), which is involved in cell growth. ALK gene mutations have been found in some types of cancer, including neuroblastoma, non‐small cell lung cancer, and anaplastic large cell lymphoma. These mutations could increase the growth of cancer cells. ALK can activate Stat3 and protects cells from cell death,^34^ which could be the reason for the association between *ALK* gene and low ctDNA level. Our study shows that ALK gene mutations in ctDNA have an association with the ctDNA concentration. Relevant studies showed that the increase in ctDNA levels might be associated with the gain of ALK gene copies.^35^, ^36^ It was discovered that ALK gene copy number gain was found in some CRC tumors, and increasing ALK gene copy was associated with poor prognosis.^35^, ^36^ The gain of ALK gene copy may have a role in resistance to anti‐EGFR therapy through cross‐talk of signaling pathways.^36^ The reason is that mutations in ALK are associated with cell proliferation, resistance to apoptosis, and enhanced DNA synthesis.^37^, ^38^ Due to the association between *ALK* gene somatic mutation and ctDNA level, the measurement of ctDNA level can help clinicians to decide if targeted therapy to ALK, such as using Crizotinib or Ceritinib, shall be given to patients.

*MSH2* and *PMS2* mutations were founded in ctDNAs^39^ in different cancer patients, including CRC.^40^ It has been shown that *MSH2* and *PMS2* alteration is associated with high microsatellite instability for tumor genomes.^39^ Microsatellite instability is found in 10% to 15% of CRCs.^41^ Mismatch repair deficient and microsatellite instability‐high, caused by *MSH2* or/and *PMS2* mutations, cause failure of apoptosis upon detection of critical DNA damage,^42^ which may increase the ctDNA levels. The gene product of *MSH2* gene plays an essential role in DNA repairing to fix errors during DNA replication in preparation for cell division, and Msh2 protein is involved in apoptosis. Mutations in the *MSH2* gene are associated with cancers, especially CRC.^43^ Mutations in MSH2 may cause DNA damage to go unrepaired, resulting in an increase in mutation frequency, and MSH2 deficiency may predispose to malignancy not only through failed repair of mismatched DNA lesions but also through the failure to engage apoptosis.^44^ The product of *PMS2* gene, forming a heterodimer with MLH1 and interacting with MSH2, is involved in DNA mismatch repair.^45^ Defects in this gene are associated with hereditary nonpolyposis colorectal cancer, with Turcot syndrome, and are a cause of supratentorial primitive neuroectodermal tumors.^46^ PMS2 has also been shown to interact with p53 and p73. PMS2 and MLH1 proteins can protect cells from cell death by enhancing apoptosis,^47^ and PMS2 deficit can cause cell death.^48^ Since cell death is related to ctDNA levels,^23^, ^24^ it is reasonable that mutations of *PMS2* are associated with ctDNA levels.

The *MAP2K1* gene encodes MEK1 protein kinase, which is involved in the RAS/MAPK signaling pathway. The RAS/MAPK signaling pathway regulates cell growth, proliferation, differentiation, migration, and apoptosis.^49^ MEK1 protein kinase appears to be essential for normal development before birth and for survival after birth, but MAP2K1 mutations were observed in many human epithelial cancers, including esophageal cancer, gastric cancer, breast cancer, and CRC.^50^ Two MEK inhibitors, Trametinib and Cobimetinib, can be used for targeted therapy to patients with *MAP2K1* mutations. Based on the association between *MAP2K1* gene mutations mutation and ctDNA level, combining measuring ctDNA level and examination of *MAP2K1* mutations can guide clinicians to apply MEK inhibitors to patients for precision medicine.

*KDR* gene encodes Kinase insert domain receptor, which functions as a type III receptor tyrosine kinase of vascular endothelial growth factor (VEGF). The KDR protein conducts regulation of VEGF‐induced endothelial proliferation, migration, sprouting, and tubular morphogenesis.^51^ Mutations of *KDR* gene are observed in many cancers such as intestinal cancer, lung cancer, and skin cancer.^52^

Using the random forest model to study the association between ctDNA concentration and gene SNVs has advantages in efficient searching for large datasets, such as genome SNVs, and reducing the variance to improve the accuracy. Because the random forest model has a lot of parameters, and then, it will overfit more easily. Therefore, during the application of the random forest model for regression, the maximum of features was set to 5 and the maximal depth was set to only 3 to reduce overfitting. To reduce the false positives and overfitting caused by the random forest model, a set of important CRC‐related genes was studied. The limited coverage of genes gives rise to potential false negatives of important SNVs.

## 4 MATERIALS AND METHODS

### 4.1 Specimen selection and grouping

A total of 85 CRC patients were recruited from January 1 to August 31, 2019, at Hunan Cancer Hospital, Changsha, China under an approved IRB protocol number: KYJJ‐2020‐132. Patients were enrolled by the following inclusion and exclusion criteria. These patients had tumors with stages I–IV, N0–N2, did not have Distant metastasis (M0), and had detectable ctDNAs. Patients with Mucinous colorectal cancer were excluded. They were selected under the following criteria—patients had the sporadic form of CRC, did not have other basic diseases, did not have an infection, and did not have other factors affecting plasma free nucleic acid concentration, such as no neo‐adjuvant treatment, chemotherapy, and/or radiation therapy. The clinical information of patients, including tumor sizes and stages, is summarized in Table 1 and their details are listed in the Dataset S1.

### 4.2 Extraction and sequencing of cell‐free nucleic acids

Tumor tissue was collected at surgery and blood samples (*n* = 85) were collected before surgery. Based on the IRB protocol for this study, patient blood samples were collected for approximately 5–10 ml and transferred into EDTA‐coated tubes. All blood samples were processed immediately or within 1 day after storage at 4°C. Plasma from blood samples was separated by centrifugation at 1600*g* for 10 min at 4°C, and a second centrifugation step was performed at 18 000*g* at room temperature to remove any remaining cellular debris. the plasma was transferred to a new tube and the supernatant is used for DNA extraction. The isolation of cfDNA was conducted by using the QIAamp Circulating Nucleic Acid kit (Qiagen Germantown, MD) and following the manufacturer's instructions. The extracted ctDNA was tested by Qbit 2.0 and analyzed by Agilent 4200 TapeStation. Eighty five samples with detailed pathology reports were selected for further sequencing studies. Some images of the quality of the isolated cfDNA from the Bioanalyzer are shown in Figure S1.

Ion Ampliseq was used for qualified cfDNA. Gene amplification was carried out according to an in‐house gene panel, including *ERBB2*, *BRAF*, *KRAS*, *NRAS*, *EGFR*, *NTRK1/2/3*, *TP53*, *KIT*, *RAF1*, *FLT1*, *FLT3*, *FLT4*, *KDR*, *PDGFRA*, *PDGFRB*, *FGFR1*, *FGFR2*, *FGFR3*, *FGFR4*, *MAP2K1*, *MAP2K2*, *mTOR*, *JAK1*, *JAK2*, *PTEN*, *PIK3CA*, *STK11*, *AKT1*, *EPCAM*, *APC*, *MUTYH*, *BMPR1A* and *SMAD4*, and the library construction was carried out for the purified product. The adapter‐ligated library was constructed with the Ion AmpliSeq Library Kit 2.0 following the manufacturer's instructions (Life Technologies). Ion S5 was used for sequencing. The effective nucleic acid fragments were selected by fragment screening according to the length of amplified target fragments. The obtained library was amplified and purified. Multiple libraries were merged for amplification. After amplification, the products were purified by magnetic beads. Finally, sequencing was conducted.

### 4.3 Analysis and quality control of sequencing data

After sequencing and base‐calling, the resulting raw fastq data were analyzed by in‐house quality control software to remove low quality reads and were then aligned to the reference human genome (hs37d5) using the Burrows‐Wheeler Aligner (BWA),^53^ and duplicate readings were marked using Sambamba tools.^54^ The raw fastq data were submitted to the National Omics Data Encyclopedia (NODE) (https://www.biosino.org/node/), and the project ID is OEP001279. Please see the sections of Data Availability and Materials for more details.

SNVs and InDels were called with GATK.^55^ The raw calls of SNVs and InDels were further filtered with the following inclusion thresholds: (1) the read depth > 200; (2) the mean mapping quality of the covering reads >30; (3) the variant quality score > 20. The list of SNVs is included in Dataset S1.

### 4.4 Experimental validation

We conducted small scale experiments to compare the gene mutations in patient primary tumors and mutations identified in ctDNA to validate if ctDNA discovered mutations are also in the primary tumor tissues. We validated mutations in primary tumors by Sanger sequencing after DNA amplification with polymerase chain reaction (PCR) to the primary tumor samples (Figure S1).

### 4.5 Data analysis and model construction

For the measured ctDNA amount of all patients, distribution models were used to fit the model, and the diagnostic analysis of these fitted models was done. The mathStatica toolkit was used for distribution parameter calculation and distribution fitting, and R was used for fitting diagnosis.

For the model of the association study, the preprocessing contains two steps to ensure the quality of the data used for the modeling process. SNVs with MAF smaller than 0.01 were dropped to target SNPs only.^56^ In the second step, SNPs with correlations larger than 0.8 were grouped together and one mutation from each cluster was picked up for the downstream analysis. For example, MSH2_p.A689D has correlation with MSH2_p.A689D, MSH6_p.E226V, PMS2_p.E473V, MSH6_p.E678K, MSH6_p.G1134E, MSH2_p.H639R, MSH6_p.K246E, MSH2_p.L505S, MSH6_p.M868V, MSH6_p.N741D, PMS2_p.P442S, MSH2_p.R373W, MLH1_p.V412I, MSH2_p.V617I, PMS2_p.Y519Ter.

The whole dataset was split into 60% training dataset and 40% test dataset. The training dataset was used to train an ensemble model of the random forest method and the MSE (mean square error) was used to measure its performance. For the random forest model, to reduce the overfitting, we adjusted hyperparameters such as maximum feature, maximum depth, sample fraction, and minimum node size. The number of independent trees was set to 100, the maximum features were 5, and the maximum depth was set to only 3 to reduce overfitting. The contribution of each mutation to the prediction was measured by a SHAP (SHapley Additive exPlanations) value on the test dataset, which is an alternative to permutation feature importance.^22^ To reduce the bias brought by the splitting of the small sample numbers, the splitting and modeling processes were repeated 1000 times. The average MSEs and average SHAP values were calculated for those 1000 models. Python libraries, scikit learn^57^ and shap,^22^ were used for the random forest model and SHAP value calculation.

### 4.6 TCGA data and survival analysis

The R package of RTCGA package (https://rtcga.github.io/RTCGA/) and the full set of somatic mutations discovered by TCGA data for the cohort of Colorectal adenocarcinoma (COADREAD) from Firehose (https://gdac.broadinstitute.org/)^58^ were used for survival analysis. The function, *kmTCGA*(), in RTCGA was used to plot Kaplan–Meier estimates of survival curves for survival data from patients with or without mutations.

## AUTHOR CONTRIBUTIONS

**Fei Bai:** Data curation (lead). **Qian Du:** Formal analysis (equal); software (lead). **Qingliang Zou:** Data curation (supporting). **Wei Dong:** Data curation (supporting). **Xinlin Lv:** Data curation (supporting). **Xiaorong Han:** Data curation (supporting). **Huijun Zhou:** Supervision (equal). **Tao Lu:** Conceptualization (equal); supervision (equal); writing – original draft (equal); writing – review and editing (equal).

## CONFLICT OF INTEREST

The authors declare no conflict of interest.

## ETHICS STATEMENT

Informed consent was voluntarily obtained from the participants who had been fully informed of the study including any of the benefits and risks involved. All procedures performed in studies involving human participants were in accordance with the institutional review board (KYJJ‐2020‐132) and independent ethics committee of Hunan Cancer Hospital, Changsha, China, and with the WMA Helsinki Declaration (as revised in 2013). Informed consent was obtained from all subjects involved in the study.

## Supporting information


**FIGURE S1.** Agilent 4200 TapeStation images for the isolated cfDNAs from five patients.
**FIGURE S2.** SNVs discovered in ctDNA were validated by Sanger sequencing after PCR amplification to corresponding primary tumor tissues. For example, here are three sanger sequencing results for samples from primary tumors.
**Dataset S1.** Patient clinical data, ctDNA concentration, and list of SNVs.

## Data Availability

The datasets presented in this study can be found in the online repository. The name of the repository is NODE (The National Omics Data Encyclopedia) (https://www.biosino.org/node/), and the project ID is OEP001279. The raw next‐generation sequencing data in fastq format can be downloaded by the following link: https://www.biosino.org/download/node/data/public/OED242886 with MD5:01d54af9e9898bfd7c50f9bef37a131c.

## References

[1] Rawla P , Sunkara T , Barsouk A . Epidemiology of colorectal cancer: incidence, mortality, survival, and risk factors. Prz Gastroenterol. 2019;14(2):89‐103. 31616522 10.5114/pg.2018.81072 PMC6791134

[2] Haupts A , Roth W , Hartmann N . Liquid biopsy in colorectal cancer: an overview of ctDNA analysis in tumour diagnostics. Pathologe. 2019;40(Suppl 3):244‐251. 31797045 10.1007/s00292-019-00698-3

[3] Ding Y , Li W , Wang K , Xu C , Hao M , Ding L . Perspectives of the application of liquid biopsy in colorectal cancer. Biomed Res Int. 2020;2020:6843180. 32258135 10.1155/2020/6843180 PMC7085834

[4] Malla M , Loree JM , Kasi PM , Parikh AR . Using circulating tumor DNA in colorectal cancer: current and evolving practices. J Clin Oncol. 2022;40(24):2846‐2857. 35839443 10.1200/JCO.21.02615 PMC9390824

[5] Dasari A , Morris VK , Allegra CJ , et al. ctDNA applications and integration in colorectal cancer: an NCI colon and Rectal‐anal task forces whitepaper. Nat Rev Clin Oncol. 2020;17(12):757‐770. 32632268 10.1038/s41571-020-0392-0 PMC7790747

[6] Bettegowda C , Sausen M , Leary RJ , et al. Detection of circulating tumor DNA in early‐ and late‐stage human malignancies. Sci Transl Med. 2014;6(224):224ra24. 10.1126/scitranslmed.3007094 PMC4017867 24553385

[7] Reece M , Saluja H , Hollington P , et al. The use of circulating tumor DNA to monitor and predict response to treatment in colorectal cancer. Front Genet. 2019;10:1118. 31824558 10.3389/fgene.2019.01118 PMC6881479

[8] Osumi H , Shinozaki E , Takeda Y , et al. Clinical relevance of circulating tumor DNA assessed through deep sequencing in patients with metastatic colorectal cancer. Cancer Med. 2019;8(1):408‐417. 30575318 10.1002/cam4.1913 PMC6346227

[9] Scholer LV , Reinert T , Orntoft MW , et al. Clinical implications of monitoring circulating tumor DNA in patients with colorectal cancer. Clin Cancer Res. 2017;23(18):5437‐5445. 28600478 10.1158/1078-0432.CCR-17-0510

[10] Calapre L , Warburton L , Millward M , Gray ES . Circulating tumour DNA (ctDNA) as a biomarker in metachronous melanoma and colorectal cancer—a case report. BMC Cancer. 2019;19(1):1109. 31727009 10.1186/s12885-019-6336-3 PMC6857141

[11] Murtaza M , Dawson SJ , Pogrebniak K , et al. Multifocal clonal evolution characterized using circulating tumour DNA in a case of metastatic breast cancer. Nat Commun. 2015;6:8760. 26530965 10.1038/ncomms9760 PMC4659935

[12] Montagut C , Argiles G , Ciardiello F , et al. Efficacy of Sym004 in patients with metastatic colorectal cancer with acquired resistance to anti‐EGFR therapy and molecularly selected by circulating tumor DNA analyses: a phase 2 randomized clinical trial. JAMA Oncol. 2018;4(4):e175245. 29423521 10.1001/jamaoncol.2017.5245 PMC5885274

[13] Evrard C , Tachon G , Randrian V , Karayan‐Tapon L , Tougeron D . Microsatellite instability: diagnosis, heterogeneity, discordance, and clinical impact in colorectal cancer. Cancers (Basel). 2019;11(10):1567. 31618962 10.3390/cancers11101567 PMC6826728

[14] Georgiadis A , Durham JN , Keefer LA , et al. Noninvasive detection of microsatellite instability and high tumor mutation burden in cancer patients treated with PD‐1 blockade. Clin Cancer Res. 2019;25(23):7024‐7034. 31506389 10.1158/1078-0432.CCR-19-1372 PMC6892397

[15] Liu Y , Du Q , Sun D , et al. Clinical applications of circulating tumor DNA in monitoring breast cancer drug resistance. Future Oncol. 2020;16:2863‐2878. 32976028 10.2217/fon-2019-0760

[16] Yang YC , Wang D , Jin L , et al. Circulating tumor DNA detectable in early‐ and late‐stage colorectal cancer patients. Biosci Rep. 2018;38(4):BSR20180322. 29914973 10.1042/BSR20180322 PMC6066652

[17] Huang K , Qu H , Zhang X , et al. Circulating tumor DNA sequencing for colorectal cancers: a comparative analysis of colon cancer and rectal cancer data. Cancer Biomark. 2019;26(3):313‐322. 31561327 10.3233/CBM-190257 PMC12826425

[18] Bhangu JS , Beer A , Mittlbock M , et al. Circulating free methylated tumor DNA markers for sensitive assessment of tumor burden and early response monitoring in patients receiving systemic chemotherapy for colorectal cancer liver metastasis. Ann Surg. 2018;268(5):894‐902. 30080722 10.1097/SLA.0000000000002901

[19] Mosebach J , Shah S , Delorme S , et al. Prognostic significance of tumor burden assessed by whole‐body magnetic resonance imaging in multiple myeloma patients treated with allogeneic stem cell transplantation. Haematologica. 2018;103(2):336‐343. 29217779 10.3324/haematol.2017.176073 PMC5792278

[20] Poklepovic AS , Carvajal RD . Prognostic value of low tumor burden in patients with melanoma. Oncology (Williston Park). 2018;32(9):e90‐e96. 30248170

[21] Lee RJ , Gremel G , Marshall A , et al. Circulating tumor DNA predicts survival in patients with resected high‐risk stage II/III melanoma. Ann Oncol. 2018;29(2):490‐496. 29112704 10.1093/annonc/mdx717 PMC5834029

[22] Lundberg SM , Lee S‐I . A Unified Approach to Interpreting Model Predictions. Proceedings of the 31st International Conference on Neural Information Processing Systems. Curran Associates Inc.; 2017:4768‐4777.

[23] Kustanovich A , Schwartz R , Peretz T , Grinshpun A . Life and death of circulating cell‐free DNA. Cancer Biol Ther. 2019;20(8):1057‐1067. 30990132 10.1080/15384047.2019.1598759 PMC6606043

[24] Oliveira KCS , Ramos IB , Silva JMC , et al. Current perspectives on circulating tumor DNA, precision medicine, and personalized clinical Management of Cancer. Mol Cancer Res. 2020;18(4):517‐528. 31996469 10.1158/1541-7786.MCR-19-0768

[25] Reinert T , Scholer LV , Thomsen R , et al. Analysis of circulating tumour DNA to monitor disease burden following colorectal cancer surgery. Gut. 2016;65(4):625‐634. 25654990 10.1136/gutjnl-2014-308859

[26] Reinert T , Henriksen TV , Christensen E , et al. Analysis of plasma cell‐free DNA by ultradeep sequencing in patients with stages I to III colorectal cancer. JAMA Oncol. 2019;5(8):1124‐1131. 31070691 10.1001/jamaoncol.2019.0528 PMC6512280

[27] Bratman SV , Yang SYC , Iafolla MAJ , et al. Personalized circulating tumor DNA analysis as a predictive biomarker in solid tumor patients treated with pembrolizumab. Nat Cancer. 2020;1(9):873‐881. 35121950 10.1038/s43018-020-0096-5

[28] Stadler JC , Belloum Y , Deitert B , et al. Current and future clinical applications of ctDNA in Immuno‐oncology. Cancer Res. 2022;82(3):349‐358. 34815256 10.1158/0008-5472.CAN-21-1718 PMC9397642

[29] Stroun M , Anker P , Maurice P , Lyautey J , Lederrey C , Beljanski M . Neoplastic characteristics of the DNA found in the plasma of cancer patients. Oncology. 1989;46(5):318‐322. 2779946 10.1159/000226740

[30] Bardelli A , Pantel K . Liquid biopsies, what we do not know (yet). Cancer Cell. 2017;31(2):172‐179. 28196593 10.1016/j.ccell.2017.01.002

[31] Cives M , Partelli S , Palmirotta R , et al. DAXX mutations as potential genomic markers of malignant evolution in small nonfunctioning pancreatic neuroendocrine tumors. Sci Rep. 2019;9(1):18614. 31819132 10.1038/s41598-019-55156-0 PMC6901561

[32] Baslan T , Kendall J , Volyanskyy K , et al. Novel insights into breast cancer copy number genetic heterogeneity revealed by single‐cell genome sequencing. Elife. 2020;9:e51480. 32401198 10.7554/eLife.51480 PMC7220379

[33] Lim Y , Kim S , Kang JK , et al. Circulating tumor DNA sequencing in colorectal cancer patients treated with first‐line chemotherapy with anti‐EGFR. Sci Rep. 2021;11(1):16333. 34381078 10.1038/s41598-021-95345-4 PMC8358023

[34] Zamo A , Chiarle R , Piva R , et al. Anaplastic lymphoma kinase (ALK) activates Stat3 and protects hematopoietic cells from cell death. Oncogene. 2002;21(7):1038‐1047. 11850821 10.1038/sj.onc.1205152

[35] Bavi P , Jehan Z , Bu R , et al. ALK gene amplification is associated with poor prognosis in colorectal carcinoma. Br J Cancer. 2013;109(10):2735‐2743. 24129244 10.1038/bjc.2013.641 PMC3833224

[36] Pietrantonio F , Maggi C , Di Bartolomeo M , et al. Gain of ALK gene copy number may predict lack of benefit from anti‐EGFR treatment in patients with advanced colorectal cancer and RAS‐RAF‐PI3KCA wild‐type status. PLoS One. 2014;9(4):e92147. 24691006 10.1371/journal.pone.0092147 PMC3972159

[37] Motegi A , Fujimoto J , Kotani M , Sakuraba H , Yamamoto T . ALK receptor tyrosine kinase promotes cell growth and neurite outgrowth. J Cell Sci. 2004;117(Pt 15):3319‐3329. 15226403 10.1242/jcs.01183

[38] Chen Y , Takita J , Choi YL , et al. Oncogenic mutations of ALK kinase in neuroblastoma. Nature. 2008;455(7215):971‐974. 18923524 10.1038/nature07399

[39] Cai Z , Wang Z , Liu C , et al. Detection of microsatellite instability from circulating tumor DNA by targeted deep sequencing. J Mol Diagn. 2020;22(7):860‐870. 32428677 10.1016/j.jmoldx.2020.04.210

[40] Wang Y , Yang L , Bao H , et al. Utility of ctDNA in predicting response to neoadjuvant chemoradiotherapy and prognosis assessment in locally advanced rectal cancer: a prospective cohort study. PLoS Med. 2021;18(8):e1003741. 34464382 10.1371/journal.pmed.1003741 PMC8407540

[41] Kang S , Na Y , Joung SY , Lee SI , Oh SC , Min BW . The significance of microsatellite instability in colorectal cancer after controlling for clinicopathological factors. Medicine (Baltimore). 2018;97(9):e0019. 29489646 10.1097/MD.0000000000010019 PMC5851768

[42] Buchler T . Microsatellite instability and metastatic colorectal cancer—a clinical perspective. Front Oncol. 2022;12:888181. 35574322 10.3389/fonc.2022.888181 PMC9097548

[43] Lee KH , Lee JS , Nam JH , et al. Promoter methylation status of hMLH1, hMSH2, and MGMT genes in colorectal cancer associated with adenoma‐carcinoma sequence. Langenbecks Arch Surg. 2011;396(7):1017‐1026. 21706233 10.1007/s00423-011-0812-9

[44] Toft NJ , Winton DJ , Kelly J , et al. Msh2 status modulates both apoptosis and mutation frequency in the murine small intestine. Proc Natl Acad Sci U S A. 1999;96(7):3911‐3915. 10097137 10.1073/pnas.96.7.3911 PMC22394

[45] Kasela M , Nystrom M , Kansikas M . PMS2 expression decrease causes severe problems in mismatch repair. Hum Mutat. 2019;40(7):904‐907. 30946512 10.1002/humu.23756 PMC6618857

[46] Liu W , Zhang D , Tan SA , Liu X , Lai J . Sigmoid colon adenocarcinoma with isolated loss of PMS2 presenting in a patient with synchronous prostate cancer with intact MMR: diagnosis and analysis of the family pedigree. Anticancer Res. 2018;38(8):4847‐4852. 30061258 10.21873/anticanres.12796

[47] Shimodaira H , Yoshioka‐Yamashita A , Kolodner RD , Wang JY . Interaction of mismatch repair protein PMS2 and the p53‐related transcription factor p73 in apoptosis response to cisplatin. Proc Natl Acad Sci U S A. 2003;100(5):2420‐2425. 12601175 10.1073/pnas.0438031100 PMC151356

[48] Marinovic‐Terzic I , Yoshioka‐Yamashita A , Shimodaira H , et al. Apoptotic function of human PMS2 compromised by the nonsynonymous single‐nucleotide polymorphic variant R20Q. Proc Natl Acad Sci U S A. 2008;105(37):13993‐13998. 18768816 10.1073/pnas.0806435105 PMC2528866

[49] Sebolt‐Leopold JS , Herrera R . Targeting the mitogen‐activated protein kinase cascade to treat cancer. Nat Rev Cancer. 2004;4(12):937‐947. 15573115 10.1038/nrc1503

[50] Choi YL , Soda M , Ueno T , et al. Oncogenic MAP2K1 mutations in human epithelial tumors. Carcinogenesis. 2012;33(5):956‐961. 22327936 10.1093/carcin/bgs099

[51] Simons M , Gordon E , Claesson‐Welsh L . Mechanisms and regulation of endothelial VEGF receptor signalling. Nat Rev Mol Cell Biol. 2016;17(10):611‐625. 27461391 10.1038/nrm.2016.87

[52] Dong G , Guo X , Fu X , et al. Potentially functional genetic variants in KDR gene as prognostic markers in patients with resected colorectal cancer. Cancer Sci. 2012;103(3):561‐568. 22182247 10.1111/j.1349-7006.2011.02194.x PMC7713614

[53] Li H , Durbin R . Fast and accurate short read alignment with burrows‐wheeler transform. Bioinformatics. 2009;25(14):1754‐1760. 19451168 10.1093/bioinformatics/btp324 PMC2705234

[54] Tarasov A , Vilella AJ , Cuppen E , Nijman IJ , Prins P . Sambamba: fast processing of NGS alignment formats. Bioinformatics. 2015;31(12):2032‐2034. 25697820 10.1093/bioinformatics/btv098 PMC4765878

[55] McKenna A , Hanna M , Banks E , et al. The genome analysis toolkit: a MapReduce framework for analyzing next‐generation DNA sequencing data. Genome Res. 2010;20(9):1297‐1303. 20644199 10.1101/gr.107524.110 PMC2928508

[56] He MM , Li Q , Yan M , et al. Variant interpretation for cancer (VIC): a computational tool for assessing clinical impacts of somatic variants. Genome Med. 2019;11(1):53. 31443733 10.1186/s13073-019-0664-4 PMC6708137

[57] Pedregosa F . Scikit‐learn: machine learning in python. J Mach Learn Res. 2011;12:2825‐2830.

[58] Deng M , Brägelmann J , Kryukov I , Saraiva‐Agostinho N , Perner S . FirebrowseR: an R client to the broad institute's firehose pipeline. Database (Oxford). 2017;2017:baw160. 28062517 10.1093/database/baw160 PMC5216271

