# Supplementary material for: The association of blood ctDNA levels to mutations of marker genes in colorectal cancer
Source: Cancer Rep (Hoboken). 2023 Feb 6;6(4):e1782. doi: 10.1002/cnr2.1782 (PMC10075297; doi:10.1002/cnr2.1782)
Supplement: Supplementary file 1 — FIGURE S1. Agilent 4200 TapeStation images for the isolated cfDNAs from five patients. FIGURE S2. SNVs discovered in ctDNA were validated by Sanger sequencing after PCR amplification to corresponding primary tumor tissues. For example, here are three sanger sequencing results for samples from primary tumors. Dataset S1. Patient clinical data, ctDNA concentration, and list of SNVs. [file CNR2-6-e1782-s001.pdf]

Supplementary Figures

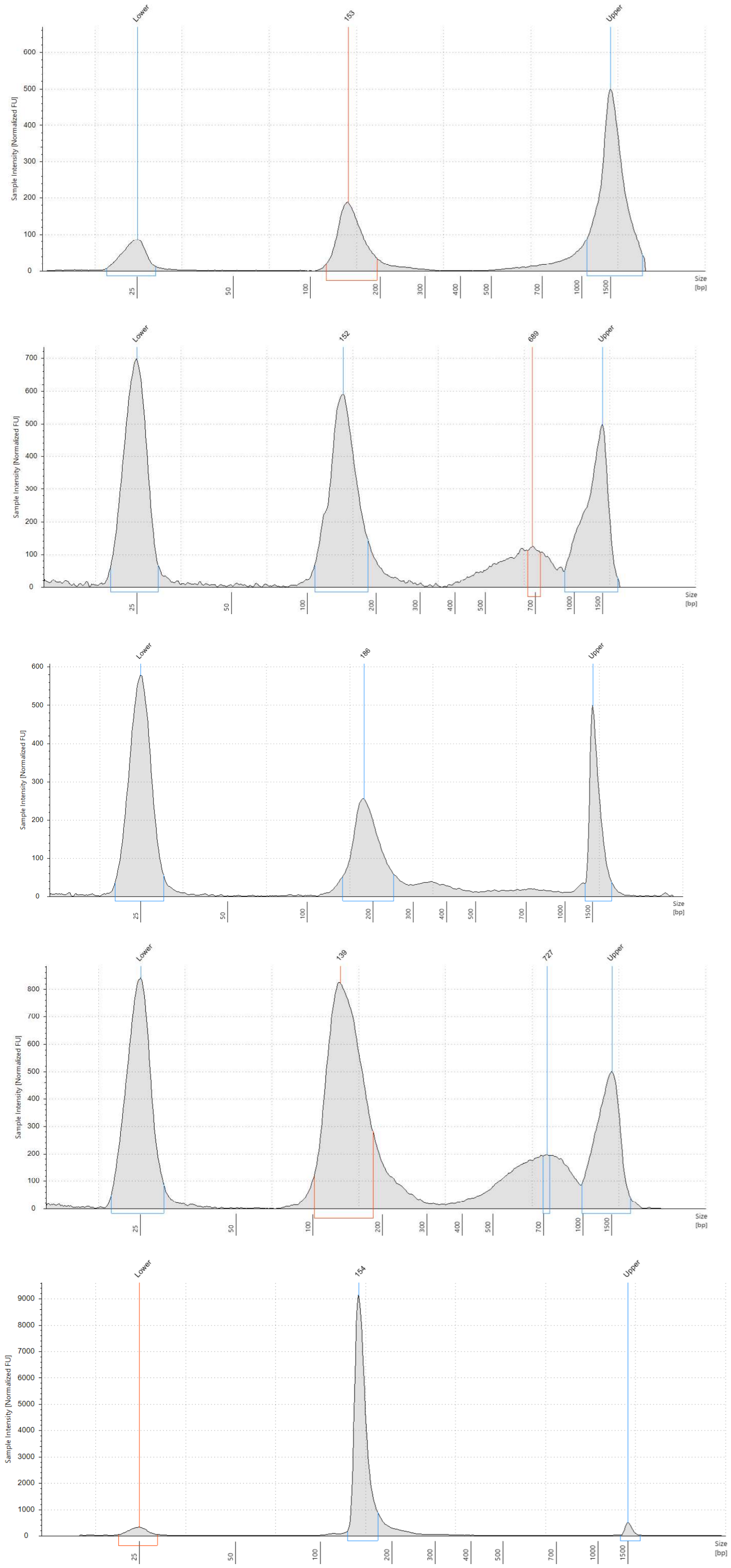

Supplementary Figure S1. Agilent 4200 TapeStation images for the isolated cfDNAs from five patients.

PMS2 rs63750871

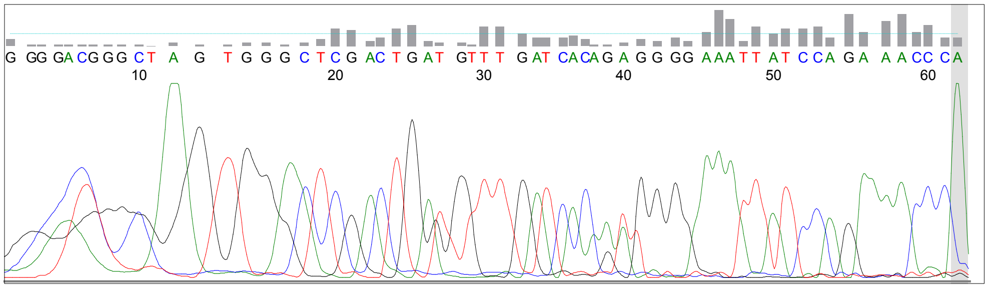

MSH6 rs267608046

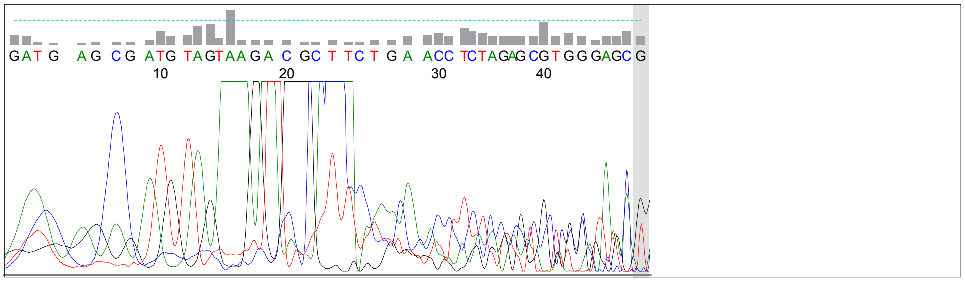

PMS2 rs63750451

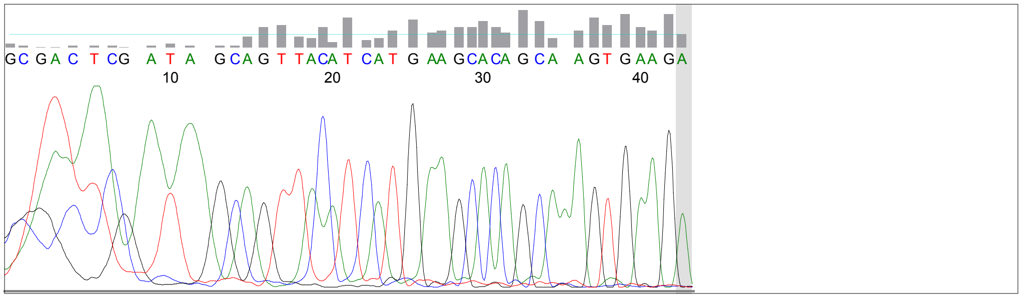

Supplementary Figure S2. SNVs discovered in ctDNA were validated by Sanger sequencing after PCR amplification to corresponding primary tumor tissues. For example, here are three sanger sequencing results for samples from the primary tumors.
